# Impact of the SARS-CoV-2 Pandemic on the Management and Prognosis of Infective Endocarditis

**DOI:** 10.3390/tropicalmed9040086

**Published:** 2024-04-17

**Authors:** Lucie Ailhaud, Robinson Gravier-Dumonceau, Florent Arregle, Sandrine Hubert, Jean-Paul Casalta, Alberto Riberi, Laetitia Tessonnier, Roch Giorgi, Gilbert Habib, Frédérique Gouriet

**Affiliations:** 1 Microbes Evolution Phylogeny and Infections (MEPHI), Assistance Publique–Hopitaux de Marseille (AP-HM), Aix Marseille University, 13005 Marseille, France; lucie-sophie.ailhaud@ap-hm.fr (L.A.); jeanpaul.casalta@ap-hm.fr (J.-P.C.); gilbert.habib@ap-hm.fr (G.H.); 2 IHU Méditerranée Infection, 13005 Marseille, France; 3 BioSTIC, Department of Biostatistics and Information and Communication Technologies, Assistance Publique–Hopitaux de Marseille (AP-HM), La Timone Hospital, 13005 Marseille, France; robinson.gravier-dumonceau@ap-hm.fr (R.G.-D.); roch.giorgi@ap-hm.fr (R.G.); 4 Department of Cardiology, Assistance Publique–Hopitaux de Marseille (AP-HM), La Timone Hospital, 13005 Marseille, France; florent.arregle@ap-hm.fr (F.A.); sandrine.hubert@ap-hm.fr (S.H.); 5 Department of Cardiac Surgery, Assistance Publique–Hopitaux de Marseille (AP-HM), La Timone Hospital, 13005 Marseille, France; alberto.riberi@ap-hm.fr; 6 Department of Nuclear Imagery, Assistance Publique–Hopitaux de Marseille (AP-HM), La Timone Hospital, 13005 Marseille, France; laetitia.tessonnier@ap-hm.fr; 7 Sciences Economiques & Sociales de la Santé & Traitement de l’Information Médicale (SESSTIM), Institut de Recherche pour le Développement (IRD), Institut National de la Santé et de la Recherche médicale (INSERM), Assistance Publique–Hopitaux de Marseille (AP-HM), Aix Marseille University, 13005 Marseille, France

**Keywords:** infective endocarditis, SARS-CoV-2, COVID-19, pandemic, intravenous drug use, management, prognosis, mortality

## Abstract

Background: Infective endocarditis (IE) is a serious condition which is difficult to diagnose and to treat, both medically and surgically. Objectives: The objective of this study was to evaluate the impact of the SARS-CoV-2 pandemic on the management of patients with IE. Methods: We conducted a single-centre retrospective study including patients hospitalized for IE during the pandemic (Group 2) compared with the same period the year before (Group 1). We compared clinical, laboratory, imagery, therapeutic, and patient outcomes between the two groups. Results: A total of 283 patients were managed for possible or definite IE (164 in Group 1 and 119 in Group 2). There were more intravenous drug-related IE patients in Group 2 (*p* = 0.009). There was no significant difference in surgery including intra-cardiac device extraction (*p* = 0.412) or time to surgery (*p* = 0.894). The one-year mortality was similar in both groups (16% versus 17.7%, *p* = 0.704). The recurrence rate was not significantly different between the two groups (5.9% in Group 2 versus 9.1% in Group 1, *p* = 0.311). Conclusions: The SARS-CoV-2 pandemic did not appear to have had a negative impact on the management of patients with IE. Maintenance of the activities of the endocarditis team within the referral centre probably contributed to this result. Nevertheless, the high proportion of intravenous drug-addicted patients in the pandemic cohort suggests that the SARS-CoV-2 pandemic had a major psychosocial impact.

## 1. Background

Infective endocarditis (IE) is a rare disease with an incidence of between 30 and 100 million cases per year worldwide. The prognosis remains poor, often as a result of delayed diagnosis. The one-year mortality rate is approximately 30%, and can be as high as 50% if surgery is not performed when indicated [1,2,3].

In March 2020, France declared a state of emergency due to the SARS-CoV-2 pandemic and ordered a strict lockdown of the population. In the context of the pandemic, organizational changes were made in many hospitals to receive and manage patients. Non-urgent surgical procedures were cancelled and medical teams were redistributed within health structures to deal with health emergencies. Intensive care units also had to modify their operations in order to increase their capacity to receive patients with severe respiratory syndrome associated with COVID-19, thus reducing the number of post-operative and emergency beds for patients requiring critical care with pathologies other than COVID-19. In addition, the SARS-CoV-2 pandemic may have increased the risk of misdiagnosis of febrile patients, as well as the risk of a delay in diagnosing IE due to reduced access to care [4,5,6,7]. In the literature, COVID-19 had an impact on the delay of diagnosis of infectious diseases such as HIV or tuberculosis with the reduction in health care access and misdiagnoses risk due to overlap of conditions [4,8]. All of these factors could potentially have had an impact on the management of patients with IE.

Recent publications have shown a decrease in the number of patients diagnosed with IE and a decrease in the number of cardiac surgical procedures performed during the SARS-CoV-2 pandemic. Our centre participated in one of these preliminary studies [8,9,10]. In our hospital reference centre for the management of IE in Marseille, we attempted to maintain our activity, through collaboration between cardiologists, infectious disease specialists, radiologists, nuclear medicine specialists, cardiac surgeons, anaesthetists, and intensive care practitioners. We maintained our weekly multidisciplinary meetings of the endocarditis team, albeit with reduced attendance. Surgical management was possible as a result of maintaining emergency cardiac surgery activity followed by dedicated intensive care beds. Despite this optimal management, following a previous study carried in our centre, we hypothesized that the SARS-CoV-2 pandemic may have had an impact, in particular, on the incidence of the disease, the delays in diagnosis and management, reduction in the number of surgical procedures carried out, and an increase in mortality during this period [9].

## 2. Objectives

The objective of this study is to describe the epidemiological, clinical, laboratory, (including microbiological), imagery, and therapeutic differences in managing patients with IE and their outcome during the SARS-CoV-2 pandemic compared with the previous year.

## 3. Methods

This is a retrospective, monocentric, descriptive study conducted in the reference cardiology department for IE at the La Timone Hospital in Marseille. Our centre infrastructure includes the emergency service, 49 operating theatres, and a medical imaging platform (scanner, ultrasound, and endoscopy). It has 288 beds, including 70 intensive care unit beds. During the study period, 26–30 (43%) intensive care beds were dedicated to COVID-19 and 90–100 (46%) medical ward beds, depending on the number of patients admitted.

### 3.1. Ethical Approval

The hospital health data were recorded on the health data access portal, PADS, under number PADS21-193. No patients objected to their data being collected during this period.

### 3.2. Patients

Patients over 16 years old managed in the Cardiology and Surgery Department of the La Timone University Hospital, Marseille treated for possible and definite IE using the modified Duke criteria/ESC criteria 2015 [1] were retrospectively included at the date of initial diagnosis and followed up for one year. They were divided into two groups:

- Group 1: Patients with possible or definite IE who began antibiotic therapy between the 1st of March 2019 and the 29th of February 2020. This corresponded to the group before the occurrence of the SARS-CoV-2 pandemic.
- Group 2: Patients with possible or definite IE who began antibiotic therapy between 1 March 2020 and 28 February 2021. This corresponded to the group during the first year of the SARS-CoV-2 pandemic.

### 3.3. Data Study

Data were collected retrospectively on an Excel spreadsheet and then anonymized for this study. No data were collected specifically for this study.

Clinical data included age, sex, Charlson comorbidity index [11], date of symptom onset, date of hospital admission, referring centre, community or nosocomial acquisition, complications (heart failure, cardiogenic shock, septic shock, embolism, intracranial haemorrhage, mycotic aneurysm, etc.), and the EuroSCORE II preoperative risk score [12]. The time to treatment was defined from the onset of symptoms to the date of hospitalization.

Microbiological and histological data included blood cultures, heart valve cultures, pacemaker or defibrillator lead cultures, bacterial serology and molecular biology (EDTA blood tests, heart valve and pacemaker or defibrillator leads), as previously described [13].

Ultrasound data included transthoracic echocardiography (TTE), transoesophageal echocardiography (TOE), which evaluated valve involvement, size of vegetation, presence of cardiac abscess, fistula or pseudoaneurysm.

Imaging data included whole body or thoracic, brain and cardiac computed tomography (CT), brain magnetic resonance imaging (MRI), cerebral arteriography, and 18F-fluorodeoxyglucose positron emission tomography-computed tomography (^18^F DG PET CT).

Therapeutic and management data included time to treatment (from the onset of symptoms to the date of hospitalization) and the indication and performance of surgical treatment.

“In-hospital mortality” was defined as the occurrence of death during the patient’s index hospitalization. “Relapse” was defined as the occurrence of a new episode of IE caused by the same bacteria as the previous episode. “Reinfection” is the occurrence of a new episode of IE caused by a different bacteria to the previous episode [14]. “Recurrence” included a relapse of the IE and reinfection. Patients were considered lost to follow-up at one year if they did not attend the one-year follow-up visit. Follow-up data included lost to follow-up at the one-year, one-year recurrence, in-hospital mortality, and one-year mortality due to *Streptococcus* spp., *Staphylococcus aureus*, and *Enterococcus* spp. IE. 

Data on the possible source of infection included panoramic dental X-ray and colonoscopy according to the bacteria in question and recommendations in guidelines [1].

### 3.4. Statistical Analysis

Continuous variables were described by their mean and standard deviation, median and interquartile range, minimum and maximum values, according to their distribution. Categorical variables were described by their number and percentage. Continuous variables were compared using the Student’s *t*-test or the Mann–Whitney U test, depending on the conditions of application. Categorical variables were compared by the chi-square test or Fisher’s test, depending on the conditions of application. Tests were performed in a two-sided situation and were considered statistically significant for *p* ≤ 0.05. Statistical analysis was performed with R software (version 4.2.0).

## 4. Results

### 4.1. Population

Between March 2019 and February 2021, 283 patients were seen for possible or definite IE in our centre. Of these patients, 164 (27 possible/136 definite) were treated in the year before the SARS-CoV-2 pandemic (Group 1) and 119 (14 possible/105 definite) in the year of the SARS-CoV-2 pandemic (Group 2). This corresponds to a 27.4% decrease in the absolute number of patients diagnosed in our centre between the two periods. Demographic and clinical data are presented in Table 1. 

Among the 119 patients, the SARS-CoV2 nasopharyngeal polymerase chain reaction (PCR) test was available for 106 patients and all were negative. The PCR test was not available for 14 patients for whom serology was performed; all were negative.

The mean age of patients managed for IE was 66.6 years in Group 1 and 65.4 years in Group 2 (*p* = 0.513). Most patients were male (sex ratio = 2.5) in both groups (*p* = 0.778). There was no significant difference in the referring centres (*p* = 0.538). Most patients were referred from an external healthcare centre (71.7% *p* = 0.538). The average Charlson score was 3.8 in Group 1 and 4.1 in Group 2 (*p* = 0.321). The mean Euroscore II was similar in both groups (*p* = 0.883).

### 4.2. Endocarditis Site

Of the 283 patients, 171 (60.4%) had native valve IE, 70 (24.7%) had prosthetic valve involvement, and 41 (14.5%) had implantable device IE. Most of the patients in this study had left heart involvement (84.5%), the majority in their aortic valve (47%). There was no significant difference between the two groups regarding the IE site. It is notable that 4/164 patients (2.4%) in Group 1 and 5/119 patients (4.2%) in Group 2 had a TAVI prosthetic aortic valve IE (Transcatheter Aortic Valve Implantation) (*p* = 0.499). 

### 4.3. Site of Infection Acquisition and Source

The source of infection did not differ between the two groups. It was community-based in 77.7% of cases (*p* = 0.484). It was care-associated in 22.3% of cases. Infection related to intravenous (IV) drug use was significantly higher in Group 2, representing 11.8% of patients, compared with 3.7% of patients in Group 1 (*p* = 0.009). 

### 4.4. Extra-Cardiac Complications of Infective Endocarditis

There was no significant difference in the occurrence of heart failure, atrioventricular block, cardiogenic or septic shock between the two groups (Table 2). Some 62.2% of patients in Group 1 and 69.7% of patients in Group 2 had a systemic embolism (*p* = 0.187), 58.5% of which were prior to treatment in Group 1 and 63.9% in Group 2 (*p* = 0.365). Cerebral haemorrhage occurred significantly more frequently in Group 2 than in Group 1 (15.1% versus 6.7%, *p* = 0.021). There was no significant difference in the occurrence of a mycotic aneurysm (*p* = 0.724) or in the occurrence of acute renal failure (*p* = 0.712). 

### 4.5. Cardiac Ultrasound Data

All patients underwent TTE. TOE was performed in 158/164 patients (96.3%) in Group 1 and 111/119 patients (93.3%) in Group 2 (*p* = 0.241). There was no significant difference in the presence of vegetation (*p* = 0.961) or its length (*p* = 0.899). There was no significant difference regarding the presence of a perivalvular complication (*p* = 0.414). A total of 27/164 patients (16.5%) in Group 1 and 24/119 patients (20.2%) in Group 2 had an annulus abscess (*p* = 0.880). Imaging data are presented in Table 3. 

### 4.6. Imaging Extension Data

There was no significant difference in imaging exams. Patients in Group 2 had significantly more cerebral tomographic scans (79.3% versus 90.8%, *p* = 0.009) and brain MRIs (22% versus 35.3%, *p* = 0.013) compared to Group 1. In addition, 118/164 patients (72%) in Group 1 and 75/119 patients (63%) in Group 2 had an F^18^ DG PET CT (*p* = 0.111).

### 4.7. Microbiological Data

Blood cultures were positive in 82.9% of cases in Group 1 and 89.9% of cases in Group 2 (*p* = 0.096). The three most common causative organisms for IE in both groups were *Streptococcus* spp. (27.2%), *Staphylococcus aureus* (25.8%), and *Enterococcus* spp. (13.4%). *Enterococcus* spp. IE accounted for 11% of cases in Group 1 and 16.8% of cases in Group 2 (*p* = 0.156). *S. aureus* IE accounted for 23.8% of cases in Group 1 and 28.6% of cases in Group 2 (*p* = 0.363). In Group 2, there were significantly more coagulase-negative *Staphylococcus* (12/164, 15.1% versus 18/119, 7.3%, *p* = 0.035) and fewer *Streptococcus* spp. (25/119, 21% versus 52/164, 31.7%, *p* = 0.046).

There were 16.5% cases of blood culture-negative endocarditis (BCNE) in Group 1 and 10.1% in Group 2 (*p* = 0.124). Regarding non-infectious IE, 1.2% of cases of endocarditis in Group 1 and 1.7% in Group 2 were of marantic origin (*p* > 0.999). Microbiological data are presented in Table 4. 

### 4.8. Clinical Management

The time to treatment did not differ significantly between the two groups (*p* = 0.077); fewer patients were treated for more than six months in Group 2 than in Group 1 (0.8% versus 6.1%) and more of them were treated for up to three months (94.1%) than in Group 1 (87.8%). Management data are presented Table 5. In Group 1, mean hospital stay was 31 (2–155) days and in Group 2, 29 (1–85) days. No statistically significant difference was noted between the two groups.

### 4.9. Surgery and Device Extraction

There was no significant difference (*p* = 0.25, *p* = 0.57) in the indication or the performance of an intervention, whether it was a valve repair, valve replacement, or an intra-cardiac device extraction (Table 5). A total of 234/283 patients (82.7%) had an indication for an interventional procedure and 172/283 patients (60.8%) had an effective procedure over the entire study period. One hundred and thirty of the 283 patients (45.9%) underwent cardiac surgery, and 34/283 patients (12%) had an intra-cardiac device removed. The median time from antibiotic therapy to completion of surgery and reasons for not performing surgery were not significantly different in the two groups. In most cases, the reason intervention was not performed was the presence of major comorbidities (71.2%).

### 4.10. Outcome and Follow-Up

The in-hospital mortality rate was 11% and the one-year mortality rate was 17%. There was no statistically significant difference between the two groups (*p* = 0.449 and *p* = 0.704, respectively). Outcome data are presented in Table 6. 

More than half of the patients were lost to follow-up at one year (57.3% in Group 1 and 52.1% in Group 2), without significant difference between the two groups (*p* = 0.384).

There was no significant difference in relapse of an IE episode or reinfection at one year between the two groups, with a rate of 7.8% over the entire period (Table 2, *p* = 0.311). Data on the occurrence of relapse or reinfection are presented in Table 7.

A total of 16/283 patients had a relapse of IE at one year. The most common causative organisms were MSSA (4/16, 25%) and *Streptococcus* spp. (4/16, 25%), followed by *Enterococcus* spp. (3/16, 18.8%). Recurrence was polymicrobial in 18.2% of cases. In addition, 3/283 patients had a reinfection at one year over the entire study period. Relapse of IE or reinfection at one year was found in IV drug users in 27.3% of cases in the entire study (6/22). There were more IV drug users presenting a relapse or reinfection at one year in Group 2 than in Group 1 (*p* = 0.054). Indeed, 4/7 patients with relapse of IE or reinfection at one year in Group 2 were IV drug users.

### 4.11. Portal of Entry

There was no significant difference in whether a dental check-up or a colonoscopy was performed between the two groups (*p* = 0.306 and *p* = 0.875, respectively).

## 5. Discussion

In our centre, we managed 283 patients with IE over a two-year period. Our population was comparable to that in the literature with regards to gender, age, and site of IE [2]. During the first year of the COVID-19 pandemic, we treated 119 patients, which is less than the average per year in our centre. In France, the diagnosis of IE represents an average of 30 cases per year per hospital [2]. Furthermore, many patients (70%) were referred by an external hospital throughout the entire study. We did not observe any significant difference in the origin of patients, which indicates that transfer from an external centre to our centre during the SARS-CoV-2 pandemic probably did not change. This is related to the fact that we are a regional referral centre for IE. However, fewer patients were diagnosed (27.4% decrease; 119 versus 164) with IE during the SARS-CoV-2 pandemic year than in the previous year. This is a trend which is also described in the literature [8,9,10] and may reflect an overall reduction in access to care.

Time to treatment is an important determinant in the prognosis of IE [1]. During the pandemic, the risk of initial misdiagnosis and lack of access to care had been raised in various case reports in the literature [3,4,5,6]. In our study, the time to treatment was not significantly different between the two groups. However, we noted that patients in Group 2 seemed to be managed more quickly than those in Group 1 (*p* = 0.077). This could be explained by the healthcare system prioritizing urgent care and decreasing basic cardiology activity by reducing the number of exams and de-programming non-urgent hospitalizations.

In our study, there was no significant difference in the site of acquisition of infection between community- and healthcare-associated IE. This was consistent with the literature [13], even though some authors have reported cases of nosocomial IE during long-term hospitalization for SARS-CoV-2 infection [15,16]. In contrast, the proportion of IE related to IV drug use was significantly higher in the pandemic year (Group 2) than in Group 1 (11.8% versus 3.7%) and also higher than that in the literature, which reported a rate of approximately 6.9% [2]. The SARS-CoV-2 pandemic had a psychosocial impact and social consequences, which may have promoted the occurrence or decompensation of psychiatric pathologies such as depression, anxiety disorders, and addictions. Moreover, patients who use intravenous drugs may have had more difficulty in accessing sterile equipment during the lockdown period [15,16,17,18,19]. Finally, paradoxically, this population may have had better and priority access to emergency care.

*Staphylococcus aureus*, *Streptococcus* spp. [18,19], and *Enterococcus* spp. was the most etiologic agent of IE which was consistent with the literature [2]. During the SARS-CoV-2 pandemic, we observed a significant increase in coagulase-negative *Staphylococcus* IE and a significant decrease in *Streptococcus* spp. IE. However, this distribution was not observed in the studies by Escola-Verge and Liu et al. [10,20]. The SARS-CoV-2 pandemic seemed to have had an impact on the diagnosis and management of cancer, with a decrease in the number of screenings, consultations, treatments, and oncological surgery [21]. In our study, despite the difficulties in accessing care, the proportion of marantic IE in the two groups was comparable to the literature, with an incidence of approximately 1% per year [13]. It is possible that it is too early to assess the impact of the delay in cancer diagnosis and oncological management on the occurrence of marantic IE, and surveillance of this data in future years could be interesting [22].

In the published series, patients with IE during the SARS-CoV-2 pandemic appeared to be significantly more severely ill than in typical times, in terms of the occurrence of symptomatic heart failure (29/39 or 74.4% versus 20/50 or 40%), cardiogenic shock (10/39 or 25.6% versus 4/50 or 8%), and septic shock (27/39 or 69.2% versus 21/50 or 42%) [20]. In our study, patients in Group 2 did not seem to have more extracardiac complications related to IE than those in Group 1, except for the occurrence of cerebral haemorrhage (*p* = 0.021). Indeed, patients in Group 2 had significantly more cerebral haemorrhages compared to Group 1. Patients in Group 2 also underwent significantly more brain imaging scans (CT and MRI) during their treatment, which may explain this result by constituting a bias. Our results underscored the necessity of performing cerebral imaging in the management of IE patient to diagnose cerebral complications. The increase in the performance of these exams was probably facilitated by the accessibility of emergency imaging due to the cancellation of non-priority exams. It should be noted that 65.4% of the patients presented an embolic event over the entire study, which is higher than reported in the literature [2]. A large number of patients (68.2%) underwent an ^18^FDG PET CT. This could explain this result, highlighting the value of this type of imaging in the context of assessing extension. There was no significant difference between the two groups regarding the performance of a TOE, an examination that could increase the risk of transmitting the SARS-CoV-2 virus [23]. Easy access to systematic SARS-CoV-2 screening in our centre was available from the beginning of the pandemic, thus facilitating the performance of TOE. Nevertheless, this result is out of line with the results of a previous study carried out in our centre, which found a 49% decrease in the performance of this test between January and April 2020 [9]. It is likely that this data was smoothed over the period of our one-year study. Moreover, patients in Group 2 did not experience significantly more cardiac complications, which is consistent with the literature [20].

Cardiac surgery and the removal of intra-cardiac devices constitute a major part of IE treatment. The prognosis of IE is severely impacted when surgery is indicated and not performed [1,2]. In their study, Nader et al. compared adult cardiac surgery activity between March and May 2020 with the same period in 2019. They observed a 57% decrease in such activity, all indications combined [24]. Nevertheless, in their study, there was a higher rate of IE surgery during the SARS-CoV-2 pandemic. These results are consistent with the study of Mikus et al., where they found a significant increase in the number of IE patients referred to their centre for surgery [25]. Our results were consistent with these data and there was no significant difference between the two groups in terms of the performance of either cardiac surgery or the removal of an intra-cardiac device. Surgical management of IE did not seem to have been impacted by the pandemic. SARS-CoV-2 infections in IE patients could have led to serious consequences for patients, delaying surgery due to risk of contagion or logistic organization [25,26]. The study of Mikus et al. noticed an increase from 16 to 19 days in the median time from endocarditis diagnosis to surgery [25]. Furthermore, in our study, there were significantly more cerebral haemorrhages in Group 2. In the case of intracranial haemorrhage, surgery must be delayed by a month according to the recommendations for IE management at the study period, which may also have delayed surgical treatment [1]. Despite these findings, there was no significant statistical difference between the two groups regarding the timescale between the introduction of antibiotic therapy and the performance of the surgical procedure with a median time for surgery of 12 days in Group 1 and 9 days in Group 2. Liu et al. compared patients with surgical IE between 2020 and 2019; delaying surgery and performing surgery did not differ between the two periods [20]. Finally, in the three studies cited above and in contrast to our results, patients appeared to have had a significantly higher preoperative risk according to their EuroSCORE II [20,24,25]. 

There was no significant difference between the two periods in terms of in-hospital mortality and one-year mortality as observed in the literature [10,20]. We noted that a large number of patients had surgery in our centre (n = 172 over two years, 60.8%).

The Belgian study previously conducted in collaboration with our centre showed discordant results, finding a hospital mortality rate of 61% during the period studied from 24 January to 30 April 2020, compared to 31% during the same period the previous year (29/47 patients versus 22/70 patients) [9]. This could be explained by the possibility of the impact of the SARS-CoV-2 pandemic during the initial strict lockdown period, which would be likely to disappear when the observation period was extended to one year, as in our study.

The overall recurrence rate of 5.3% was similar to that found in the literature (between 2% and 6%) [1]. It should be noted that relapse or reinfection at one year was more often associated with IV drug use in Group 2 than in Group 1. In Group 2, most patients with relapse or reinfection at one year were IV drug users. The SARS-CoV-2 pandemic may have had a major psychosocial impact on this population. Moreover, IV drug addiction is known to be a major risk factor for relapse [1]. Including addictologists in the endocarditis team would be an interesting perspective [27].

There was no significant difference in patients lost to follow-up at one year, which concerns most of them, for both periods. This shows the difficulty of long-term patient follow-up. The follow-up of Group 1 was during the pandemic period. Although we may have expected to see a difference in terms of mortality, recurrence, or the portal of entry compared to Group 2 because of this overlap, we did not. This could constitute a bias, but reinforced the fact that the SARS-CoV-2 pandemic did not have a significant impact upon the follow-up and outcomes of IE patients, due the maintenance of our usual management procedures.

In the literature, an endocarditis team available in a hospital has been shown to be useful [1]. In some centres, cancelling endocarditis team meetings during the SARS-CoV-2 pandemic may have had an impact on the management of IE [10]. In our centre, maintaining a multidisciplinary approach through the endocarditis team meetings probably allowed us to maintain the quality of care for these complexes. In the Liu et al. study, the endocarditis team meetings during the pandemic were maintained. Their results were consistent with ours, particularly on surgery therapeutic and mortality data [20]. 

## 6. Study Limitations

This is a retrospective monocentric study, although most patients were referred from hospitals outside the IE referral centre. The follow-up period of Group 1 patients was during the SARS-CoV-2 pandemic, which may constitute a bias. We also did not specifically study the timescale to performing surgery or removing devices in the context of isolation for SARS-CoV-2 positivity. Between 1 March 2020 to 28 February 2021, in our centre, 43% of intensive care beds were dedicated to COVID-19 and 46% of medical ward beds depending on the number of patients admitted. It is possible that our results are not applicable to other hospitals who were admitting mostly COVID-19 patients where patients with IE may have suffered a delay in terms of diagnosis and treatment.

## 7. Conclusions

The SARS-CoV-2 pandemic did not seem to have had a negative impact on the treatment of patients in our centre and their evolution at one year over the study period. The maintenance of the endocarditis team during the SARS-CoV-2 pandemic probably played a role in this, by allowing optimal management of patients. This reinforces the importance of having a referral unit for IE within a hospital centre. Furthermore, the high proportion of intravenous drug-addicted patients in our cohort in the first year of the pandemic suggests that the SARS-CoV-2 pandemic had a major psychosocial impact on the population.

## Figures and Tables

**Table 1:** Demographic and clinical data for 283 patients, La Timone Hospital, Marseille, stratified pre- and post-COVID-19 pandemic.

Characteristic	Group 1 N = 164 ^a^	Group 2 N = 119 ^a^	Total N = 283 ^a^	*p* Value
Population				
Age				0.513 ^b^
Mean [±SD]	66.6 [±14.9]	65.4 [±15.5]	66.1 [±15.1]	
Min–Max	16–89	24–91	16–91	
Male gender	116 (70.7%)	86 (72.3%)	202 (71.4%)	0.778 ^c^
Patient origin				0.538 ^c^
Timone University Hospital of Marseille	39 (23.8%)	24 (20.2%)	63 (22.3%)	
North University Hospital, Marseille	8 (4.9%)	9 (7.6%)	17 (6.0%)	
Other external centre	117 (71.3%)	86 (72.3%)	203 (71.7%)	
Charlson’s Index				0.321 ^b^
Mean [±SD]	3.8 [±2.6]	4.1 [±2.6]	3.9 [±2.6]	
Min–Max	0–12	0–10	0–12	
Euroscore II (%)				0.883 ^b^
Mean [±SD]	8.4 [±9.6]	8.3 [±8.8]	8.4 [±9.3]	
IE according to ESC 2015 criteria				
Definite IE	136 (82.9%)	105 (88.2%)	241 (85.2%)	0.215 ^c^
Possible IE	27 (16.5%)	14 (11.8%)	41 (14.5%)	0.268 ^c^
Endocarditis site				
Left side	133 (81.1%)	106 (89.1%)	239 (84.5%)	0.067 ^c^
Aortic	74 (45.1%)	59 (49.6%)	133 (47.0%)	0.458 ^c^
Mitral	60 (36.6%)	57 (47.9%)	117 (41.3%)	0.056 ^c^
Right side	40 (24.4%)	23 (19.3%)	63 (22.3%)	0.312 ^c^
Tricuspid	13 (7.9%)	5 (4.2%)	18 (6.4%)	0.205 ^c^
Pulmonary	2 (1.2%)	1 (0.8%)	3 (1.1%)	>0.999 ^d^
Native valve	93 (56.7%)	78 (65.5%)	171 (60.4%)	0.133 ^c^
Prosthetic valve	43 (26.2%)	27 (22.7%)	70 (24.7%)	0.497 ^c^
Including TAVI	4 (2.4%)	5 (4.2%)	9 (3.2%)	0.499 ^d^
ICD/PM	25 (15.2%)	16 (13.4%)	41 (14.5%)	0.671 ^c^
Source of infection				
Community-acquired infection	129 (79.17%)	90 (76.3%)	219 (77.7%)	0.484 ^c^
Healthcare-associated infection	34 (20.9%)	29 (23.7%)	63 (22.3%)	
Including drug-related infection IV	6 (3.7%)	14 (11.8%)	20 (7.1%)	0.009 ^c^
				0.468 ^c^

^a^ n (%); ^b^ Student’s *t* test; ^c^ Chi-square test; ^d^ Fisher’s exact test. IE: infective endocarditis, TAVI: trans-aortic valve implementation, IV: intravenous, ICD = implantable cardioverter-defibrillator, PM = pacemaker.

**Table 2:** Complications of IE in 283 patients, La Timone Hospital, Marseille, stratified pre- and post-COVID-19 pandemic.

Characteristic	Group 1, N = 164 ^a^	Group 2, N = 119 ^a^	Total, N = 283 ^a^	*p* Value
Extra-cardiac complications				
Heart failure	48 (29.3%)	32 (26.9%)	80 (28.3%)	0.661 ^b^
Atrioventricular block	14 (8.5%)	5 (4.2%)	19 (6.7%)	0.150 ^b^
Cardiogenic shock	10 (6.1%)	8 (6.7%)	18 (6.4%)	0.832 ^b^
Septic shock	5 (3.0%)	7 (5.9%)	12 (4.2%)	0.243 ^b^
Total systemic embolism	102 (62.2%)	83 (69.7%)	185 (65.4%)	0.187 ^b^
Systemic embolism before treatment	96 (58.5%)	76 (63.9%)	172 (60.8%)	0.365 ^b^
Cerebral embolism	42 (25.6%)	37 (31.1%)	79 (27.9%)	0.310 ^b^
Spondylodiscitis	22 (13.4%)	21 (17.6%)	43 (15.2%)	0.328 ^b^
Cerebral haemorrhage	11 (6.7%)	18 (15.1%)	29 (10.2%)	0.021 ^b^
Mycotic aneurysm of the CNS	4 (2.4%)	4 (3.4%)	8 (2.8%)	0.724 ^c^
Acute renal failure	60 (36.6%)	41 (34.5%)	101 (35.7%)	0.712 ^b^

^a^ n (%); ^b^ Chi-square test; ^c^ Fisher’s exact test.

**Table 3:** Imaging data in 283 patients, La Timone Hospital, Marseille, stratified pre- and post-COVID-19 pandemic.

Characteristic	Group 1, N = 164 ^a^	Group 2, N = 119 ^a^	Total, N = 283 ^a^	*p* Value
Ultrasound data				
TTE	164 (100.0%)	119 (100.0%)	283 (100.0%)	
TOE	158 (96.3%)	111 (93.3%)	269 (95.1%)	0.241 ^b^
Presence of vegetation	125 (76.2%)	91 (76.5%)	216 (76.3%)	0.961 ^b^
Length of vegetation				0.899 ^b^
Not known	76 (46.3%)	52 (43.7%)	128 (45.2%)	
<10 mm	15 (9.1%)	10 (8.4%)	25 (8.8%)	
10–15 mm	25 (15.2%)	22 (18.5%)	47 (16.6%)	
>15 mm	48 (29.3%)	35 (29.4%)	83 (29.3%)	
Perivalvular complication	32 (19.5%)	28 (23.5%)	60 (21.2%)	0.414 ^b^
Annular abscess	27 (16.5%)	24 (20.2%)	51 (18.0%)	0.423 ^b^
Aortic annular abscess	17 (10.4%)	13 (10.9%)	30 (10.6%)	0.880 ^b^
Mitral annular abscess	11 (6.7%)	11 (9.2%)	22 (7.8%)	0.431 ^b^
Pseudoaneurysm	3 (1.8%)	5 (4.2%)	8 (2.8%)	0.287 ^c^
Fistula	1 (0.6%)	2 (1.7%)	3 (1.1%)	0.574 ^c^
Assessment of extension				
Whole body or thoracic CT	149 (90.9%)	105 (88.2%)	254 (89.8%)	0.473 ^b^
Brain CT	130 (79.3%)	108 (90.8%)	238 (84.1%)	0.009 ^b^
Cardiac CT	93 (56.7%)	73 (61.3%)	166 (58.7%)	0.434 ^b^
Brain MRI	36 (22.0%)	42 (35.3%)	78 (27.6%)	0.013 ^b^
^18^FDG PET CT	118 (72.0%)	75 (63.0%)	193 (68.2%)	0.111 ^b^

^a^ n (%); ^b^ Chi-square test; ^c^ Fisher’s exact test. TTE: transthoracic echocardiography, TOE: trans-oesophageal echocardiography.

**Table 4:** Microbiological data in 283 patients, La Timone Hospital, Marseille, stratified pre- and post-COVID-19 pandemic.

Characteristic	Group 1, N = 164 ^a^	Group 2, N = 119 ^a^	Total, N = 283 ^a^	*p* Value
Microbiological data				
Positive blood cultures	136 (82.9%)	107 (89.9%)	243 (85.9%)	0.096 ^b^
Positive valve histopathology	37 (57.8%)	27 (51.9%)	64 (55.2%)	0.526 ^b^
Positive valve/lead culture	22 (24.4%)	9 (13.8%)	31 (20.0%)	0.104 ^b^
Positive valve/lead PCR	46 (51.7%)	28 (43.1%)	74 (48.1%)	0.291 ^b^
Germs				
*Staphylococcus aureus*	39 (23.8%)	34 (28.6%)	73 (25.8%)	0.363 ^b^
Coagulase-negative *Staphylococcus*	12 (7.3%)	18 (15.1%)	30 (10.6%)	0.035 ^b^
*Streptococcus* spp.	52 (31.7%)	25 (21.0%)	77 (27.2%)	0.046 ^b^
*Enterococcus* spp.	18 (11.0%)	20 (16.8%)	38 (13.4%)	0.156 ^b^
HACCEK	5 (3.0%)	2 (1.7%)	7 (2.5%)	0.703 ^c^
Non-HACCEK Gram-negative bacillus	8 (4.9%)	8 (6.7%)	16 (5.7%)	0.507 ^b^
Gram-positive bacillus	3 (1.8%)	1 (0.8%)	4 (1.4%)	0.641 ^c^
Other bacteria	0 (0.0%)	2 (1.7%)	2 (0.7%)	0.176 ^c^
Fungi	3 (1.8%)	2 (1.7%)	5 (1.8%)	>0.999 ^c^
BCNE	27 (16.5%)	12 (10.1%)	39 (13.8%)	0.124 ^b^
*Coxiella burnetii*	6 (3.7%)	1 (0.8%)	7 (2.5%)	0.245 ^c^
*Bartonella* sp.	2 (1.2%)	0 (0.0%)	2 (0.7%)	0.511 ^c^
*Tropheryma whipplei*	0 (0.0%)	2 (1.7%)	2 (0.7%)	0.176 ^c^
Truly negative BCNE	19 (11.6%)	9 (7.6%)	28 (9.9%)	0.263 ^b^
Marantic	2 (1.2%)	2 (1.7%)	4 (1.4%)	>0.999 ^c^

^a^ n (%); ^b^ Chi-square test; ^c^ Fisher’s exact test. BCNE = blood culture-negative endocarditis, HACCEK = *Haemophilus*, *Actinobacillus* *actinomycetemcomitans*, *Cardiobacterium* *hominis*, *Capnocytophaga* spp. *Eikenella corrodens*, and *Kingella kingae*.

**Table 5:** Management data in 283 IE patients, La Timone Hospital, Marseille, stratified pre- and post-COVID-19 pandemic.

Characteristic	Group 1, N = 164 ^a^	Group 2, N = 119 ^a^	Total, N = 283 ^a^	*p* Value
Management of IE				
Time to adequate antibiotic therapy				0.077 ^b^
<1 month	121 (74.2%)	87 (73.1%)	208 (73.8%)	
1–3 months	23 (14.1%)	25 (21.0%)	48 (17.0%)	
3–6 months	9 (5.5%)	6 (5.0%)	15 (5.3%)	
>6 months	10 (6.1%)	1 (0.8%)	11 (3.9%)	
Unknown	1	0	1	
Intervention				
Indicated intervention	132 (80.5%)	102 (85.7%)	234 (82.7%)	0.251 ^c^
Effective intervention	103 (62.8%)	69 (58.0%)	172 (60.8%)	0.412 ^c^
Surgery	73 (44.5%)	57 (47.9%)	130 (45.9%)	0.572 ^c^
Cardiac device extraction	23 (14.0%)	11 (9.2%)	34 (12.0%)	0.222 ^c^
Reason for no intervention				0.629 ^b^
Patient refusal	1 (2.3%)	4 (10.8%)	5 (6.2%)	
Multiple comorbidities	31 (72.1%)	26 (70.3%)	57 (71.2%)	
Died before surgery	5 (11.6%)	4 (10.8%)	9 (11.2%)	
Doctor’s choice	4 (9.3%)	2 (5.4%)	6 (7.5%)	
Time to intervention (days)				0.894 ^d^
Median (IQR)	12 (7–20)	9 (6–14)	12 (7–18)	
Range	0–180	1–210	0–210	

^a^ n (%); ^b^ Fisher’s exact test; ^c^ Chi-square test; ^d^ Student’s *t* test.

**Table 6:** Outcome and follow-up data in 283 IE patients, La Timone Hospital, Marseille, stratified pre- and post-COVID-19 pandemic.

Characteristic	Group 1, N = 164 ^a^	Group 2, N = 119 ^a^	Total, N = 283 ^a^	*p* Value
Outcome				
Hospital mortality	16 (9.8%)	15 (12.6%)	31 (11.0%)	0.449 ^b^
Patients who died at one year	29 (17.7%)	19 (16.0%)	48 (17.0%)	0.704 ^b^
Relapse or reinfection at one year	15 (9.1%)	7 (5.9%)	22 (7.8%)	0.311 ^b^
Lost to follow-up at one year	66 (40.2%)	43 (36.1%)	109 (38.5%)	0.384 ^b^
Portal of entry assessment				
Dental check-up	20 (12.2%)	10 (8.4%)	30 (10.6%)	0.306 ^b^
Colonoscopy	21 (12.8%)	16 (13.4%)	37 (13.1%)	0.875 ^b^

^a^ n (%); ^b^ chi-square test.

**Table 7:** Relapse and reinfection at one year in 283 IE patients, La Timone Hospital, Marseille, stratified pre- and post-COVID-19 pandemic.

Characteristic	Group 1 N = 15 ^a^	Group 2 N = 7 ^a^	Total N = 22 ^a^	*p* Value
Relapse or reinfection at one year	15 (100%)	7 (100%)	22 (100%)	0.311 ^b^
Relapse	12 (80.0%)	4 (57.1%)	16 (72.7%)	0.334 ^c^
MSSA	3 (25.0%)	1 (25.0%)	4 (25.0%)	>0.999 ^c^
Coagulase-negative *Staphylococcus*	0 (0.0%)	1 (25.0%)	1 (6.2%)	0.250 ^c^
*Streptococcus* spp.	4 (33.3%)	0 (0.0%)	4 (25.0%)	0.516 ^c^
*Enterococcus* spp.	2 (16.7%)	1 (25.0%)	3 (18.8%)	>0.999 ^c^
Gram-negative bacilli	2 (16.7%)	0 (0.0%)	2 (12.5%)	>0.999 ^c^
Fungi	1 (8.3%)	1 (25.0%)	2 (12.5%)	0.450 ^c^
Polymicrobial relapse	3 (20.0%)	1 (14.3%)	4 (18.2%)	>0.999 ^c^
Reinfection	1 (6.7%)	2 (28.6%)	3 (13.6%)	0.227 ^c^
Not known	2 (13.3%)	1 (14.3%)	3 (13.6%)	>0.999 ^c^
IV drug users	2 (13.3%)	4 (57.1%)	6 (27.3%)	0.054 ^c^

^a^ n (%); ^b^ chi-square test; ^c^ Fisher’s exact test. MSSA: methicillin-sensible staphylococcus aureus, IV = intravenous.

## Data Availability

The data may be accessible upon reasonable request.

## Abbreviations

IE = infective endocarditis, MSSA = Methicillin-Susceptible Staphylococcus Aureus, IAD = implantable automatic defibrillator, PM = pacemaker, TAVI = transcatheter aortic valve implantation, BCNE = blood culture-negative endocarditis, IV = intravenous, TTE = transthoracic echocardiography, TOE = transoesophageal echocardiography, HACCEK = *Haemophilus*, *Actinobacillus actinomycetemcomitans*, *Cardiobacterium hominis*, *Capnocytophaga* spp. *Eikenella corrodens*, and *Kingella kingae*.
